# Supplementary material for: The effects of genital myiasis on the diversity of the vaginal microbiota in female Bactrian camels
Source: BMC Vet Res. 2022 Mar 5;18:87. doi: 10.1186/s12917-022-03189-5 (PMC8897907; doi:10.1186/s12917-022-03189-5)
Supplement: Supplementary file 5 — Additional file 5. [file 12917_2022_3189_MOESM5_ESM.zip › MPL201709200_16s_yy/Treat1/css/js_css/pdfmake-master/examples/pdfs/styling_inlines.pdf]

This is a header (whole paragraph uses the same header style)

It is however possible to provide an array of texts to the paragraph (instead of a single string) and have **a better** control over it.

Each inline can be styled *independently* then.

Mixing named styles and style-overrides

We can also mix named-styles and style-overrides at both paragraph and inline level. For example, this paragraph uses the "bigger" style, which changes fontSize to 15 and sets italics to true. Texts are not italics though. It's because we've overriden italics back to false at the paragraph level.

We can also change the style of a single inline. Let's use a named style called header: **like here.**
It got bigger and bold.

OK, now we're going to mix named styles and style-overrides at the inline level. We'll use header style (it makes texts bigger and bold), but we'll override bold back to false: wow! it works!

Make sure to take a look into the sources to understand what's going on here.
